# Maternal hemoglobin concentration during pregnancy and risk of infant leukaemia: a children's oncology group study

**DOI:** 10.1038/sj.bjc.6603388

**Published:** 2006-09-26

**Authors:** A M Peters, C K Blair, M R Verneris, J P Neglia, L L Robison, L G Spector, G H Reaman, C A Felix, J A Ross

**Affiliations:** 1 Department of Pediatrics, University of Minnesota, 420 Delaware St. SE, Minneapolis, MN 55455, USA; 2 Children's Oncology Group, 440 E. Huntington Drive, Suite 300, Arcadia, CA 91006, USA; 3 St. Jude's Research Hospital, 332 North Lauderdale St., Memphis, TN 38105, USA; 4 Children's Oncology Group—Chair's Office, 4600 East West Highway, Bethesda, MD 20814, USA; 5 Children's Hospital of Philadelphia, 3516 Civic Center Boulevard, Philadelphia, PA 19104, USA

**Keywords:** anaemia, infant, leukaemia, etiology

## Abstract

In contrast to the positive association found in three studies between maternal anaemia during pregnancy and childhood leukaemia, no such association was found in infant leukaemia (odds ratio 0.85, 95% confidence interval 0.53–1.37).

Maternal anaemia during pregnancy has been positively associated with childhood leukaemia in three studies (Petridou *et al*, 1997; Roman *et al*, 1997; Roman *et al*, 2005). Given the positive associations between maternal anaemia and high birth weight (Steer *et al*, 1995; Malhotra *et al*, 2002; Chang *et al*, 2003) and high birth weight and infant leukaemia (Hjalgrim *et al*, 2003), we hypothesised that there may also be a positive association between maternal anaemia during pregnancy and infant leukaemia. We used data from the Children's Oncology Group's (COG) protocol AE24, a North American case–control study designed to evaluate possible risk factors for infant leukaemia. This is the first study limiting the evaluation of maternal anaemia to its effects on infant leukaemia, a distinct type of childhood leukaemia.

## METHODS AND MATERIALS

The human subject protection committees of each participating COG institution approved the study protocol. Eligibility criteria for cases included: (1) diagnosis with acute leukaemia in the first year of life between 1 January 1996 and 20 August 2002; (2) an English-speaking biological mother available for a telephone interview and (3) no Down syndrome. Controls were identified and contacted by random digit dialling (RDD) (for more details see Spector *et al*, 2005).

Mothers were interviewed on demographic aspects, possible exposures and birth characteristics of the index child. Separate informed consent was obtained for access to the mother's medical records for 6 months prior to the index pregnancy up through 1 week after the birth or discharge from the hospital. Maternal haemoglobin concentrations, red cell indices and the date the blood was drawn were abstracted from the medical record. Reports of leukaemia cell cytogenetic and molecular abnormalities were obtained from the treating COG institution.

A total of 348 eligible cases were identified at 126 participating COG institutions. Maternal interviews were completed for 240 cases (69%). Using RDD, a total of 430 households were identified with a child eligible as a control. Interviews were completed for 255 of the 430 eligible controls, an RDD response rate of 59%. Medical records were obtained for 236 case mothers and 215 control mothers.

Statistical evaluation was performed using SAS version 9.1, with odds ratios (ORs) calculated using unconditional logistic regression, for all infant leukaemia cases and leukaemia subtypes defined by leukaemia type (acute lymphoblastic leukaemia (ALL) *vs* acute myeloid leukaemia (AML)) and the presence of *MLL* gene rearrangements (*MLL*+ *vs MLL*−). Potential confounders evaluated were maternal age at time of index child's birth, maternal race and education, household income, smoking during pregnancy, birth order of the child, birth weight and child gender.

All mothers with an available haemoglobin value were included in the analysis. Anaemia was defined as a haemoglobin concentration of <11 g dl^−1^ (World Health Organization (WHO) classification, WHO expert committee, 1965), but a stricter definition was also used (haemoglobin <10 g dl^−1^). Haemoglobin concentration was also analysed as both a categorical and continuous exposure.

A more specific analysis was performed using the physiologic anaemia of pregnancy, a normal drop in haemoglobin due to plasma volume expansion, as the exposure of interest. This anaemia, which presents in the second or third trimester, is intriguing because it is associated with the highest average birth weight (Steer, 2000). Physiologic anaemia of pregnancy was defined as haemoglobin of <11 g dl^−1^ that developed in the second or third trimester. First trimester anaemia is likely due to nutritional deficiency and results in poor foetal outcomes including low birth weight (Hamalainen *et al*, 2003). Mothers who were anaemic in the first trimester were excluded from this analysis (four cases, three controls). Mothers with no haemoglobin values from the second or third trimester were also excluded (54 cases, 32 controls).

## RESULTS

Haemoglobin values were available for 229 cases and 207 controls; 48 case mothers were anaemic compared to 47 controls. We found no evidence that a maternal haemoglobin concentration of <11 g dl^−1^ at any time during pregnancy was associated with increased odds of infant leukaemia (data not shown). Applying more stringent criteria for anaemia (haemoglobin <10 g dl^−1^), dividing the haemoglobin concentration into categories (mild *vs* severe anaemia), and evaluating haemoglobin concentration as a continuous variable, also did not affect the results (data not shown).

Further evaluation was performed for the physiologic anaemia of pregnancy. A total of 178 case and 180 control mothers were included in this analysis. Of these cases, 115 were ALL (71 MLL+) and 63 were AML (21 MLL+).

Baseline characteristics were assessed for the 358 women included in this analysis (see Table 1). There was a significant difference in maternal race; more case mothers were Hispanic than control mothers (5.6 *vs* 1.7%, *P*=0.004). There was also a statistically significant difference in maternal age and birth order of the index child (see Table 1).

Overall, the unadjusted OR for physiologic anaemia of pregnancy and risk of infant leukaemia was 0.85 (95% confidence interval (CI) 0.53–1.37) (see Table 2). The association remained non-significant when cases were divided into AML (*n*=63) and ALL (*n*=115), with ORs of 0.64 (95% CI 0.32–1.30) and 0.98 (95% CI 0.58–1.66), respectively. There was no association when cases were stratified by *MLL* status (MLL+ OR 0.77; 95% CI 0.42–1.40, MLL− OR 0.88; 95% CI 0.45–1.73). There was also no association when the data were adjusted for maternal race and birth order. Other variables did not significantly alter the association and were not included in the final model.

## DISCUSSION

We found no evidence for increased odds of infant leukaemia in the offspring of mothers with a haemoglobin concentration of <11 g dl^−1^ during pregnancy. Three previous studies found a positive association between maternal anaemia and childhood leukaemia. A case–control study of 153 cases of acute leukaemia diagnosed in children from birth to 14 years of age in Greece from 1993 to 1994 used maternal report of anaemia during pregnancy and found a positive association (OR 2.6; 95% CI 1.39–4.86) (Petridou *et al*, 1997). Another group analysing leukaemia cases qin England found an association in two case–control studies (Roman *et al*, 1997, 2005). The more recent study compared 1485 children with leukaemia with 4864 controls. They found an OR of 2.6 (95% CI 1.7–4.1) for AML in children whose mothers had haemoglobin of <10 g dl^−1^ during pregnancy. This included 25 children with AML whose mothers were anaemic. Interestingly, this diagnostic group had a high median birth weight (3656 g), which was heavier than any other group in the analysis (Roman *et al*, 2005).

Our findings cannot confirm these studies as these covered all childhood leukaemias whereas our study concerned only infant leukaemia. It is possible the association seen previously does not exist for infant leukaemia. Also, in our North American patient population, prenatal care may differ from those in the earlier European studies. Our study subjects had essentially uniform use of prenatal vitamins, iron and folate supplementation.

One weakness of our study is the lack of power. At 80% power, the study is only capable of detecting an OR of about 2.0 or greater. Subtler increases could be missed. A potential source of bias is the selective enrolment of eligible RDD identified controls (59% field response rate). There were significant differences between cases and controls with respect to maternal race, maternal age and birth order (see Table 1). Although adjustment for these factors did not appreciably affect the risk estimates, we cannot rule out residual confounding. A strength of this study is the use of medical records to determine haemoglobin concentrations, which eliminates maternal recall bias.

Despite the negative findings for this study, the plausible associations of maternal anaemia during pregnancy, high birth weight and childhood leukaemia remain an intriguing area for aetiology research. This study was inadequately powered to detect subtle associations and should be repeated with a larger sample size.

## Figures and Tables

**Table 1:** Baseline characteristics of cases and controls with medical record data

	**Cases (*n*=178) (%)**	**Controls (*n*=180) (%)**	***P*-value**
*Maternal education*			
⩽High school	36.5	27.8	0.08
>High school	32.0	36.7	
⩾College graduate	31.5	35.6	
			
*Household income*			
<$30 000	36.2	27.8	0.09
$30 000–$75 000	46.3	48.3	
>$75 000	17.5	23.9	
			
*Maternal race*			
White	79.2	86.7	0.004
Black	2.3	5.0	
Hispanic	11.2	3.3	
Other	7.3	5.0	
			
Smoked during pregnancy	18	20.6	0.53
			
Maternal age at birth of index child	28.7	30.2	0.007
			
			
Average birth weight	3503 g	3467 g	0.52
			
Average birth order (no. of previous live births)	0.84	1.1	0.02
			
Average minimum hemoglobin concentration	11.69	11.60	0.42

**Table 2:** Association between anaemia during pregnancy (haemoglobin concentration <11 g dl^−1^) and infant leukaemia

	**Odds ratio (95% CI) unadjusted**	**Adjusted^a^ ODDS ratio (95% CI)**
Overall leukaemia (*n*=178)	0.85 (0.53–1.37)	0.93 (0.57–1.53)
AML (*n*=63)	0.64 (0.32–1.30)	0.67 (0.32–1.37)
ALL (*n*=115)	0.98 (0.58–1.66)	1.14 (0.65–2.01)
MLL+ (*n*=92)	0.77 (0.42–1.40)	0.88 (0.47–1.63)
MLL− (*n*=62)	0.88 (0.45–1.73)	0.96 (0.48–1.91)
AML/MLL+ (*n*=21)	0.46 (0.13–1.64)	0.57 (0.16–2.07)
AML/MLL− (*n*=29)	0.72 (0.28–1.88)	0.70 (0.26–1.89)
ALL/MLL+ (*n*=71)	0.87 (0.46–1.65)	0.98 (0.50–1.91)
ALL/MLL− (*n*=33)	1.04 (0.45–2.39)	1.33 (0.55–3.24)

a Association is adjusted for maternal race (white, black, Hispanic and other) and birth order (number of live births prior to index child). Maternal education (⩽HS, >HS <college grad, ⩾college grad), household income (<$30 000, $30 000–$75 000, >$75 000), maternal age at time of index child's birth, child gender, smoking during pregnancy and birth weight were not significant confounders and are not included in the adjusted model.

## References

[bib1] Chang S, O'Brien K, Nathanson M, Mancini J, Witter F (2003) Hemoglobin concentrations influence birth outcomes in pregnant African-American adolescents. J Nutr 133: 2348–2355 12840205 10.1093/jn/133.7.2348

[bib2] Hamalainen H, Hakkarainen K, Heinonen S (2003) Anaemia in the first but not second or third trimester is a risk factor for low birth weight. Clin Nutr 22: 271–275 12765667 10.1016/s0261-5614(02)00209-1

[bib3] Hjalgrim LL, Westergaard T, Rostgaard K, Schmiegelow K, Melbye M, Hjalgrim H, Engels EA (2003) Birth weight as a risk factor for childhood leukemia: a meta-analysis of 18 epidemiologic studies. Am J Epidemiol 158: 724–735 14561661 10.1093/aje/kwg210

[bib4] Malhotra M, Sharma J, Batra S, Sharma S, Murthy N, Arora R (2002) Maternal and perinatal outcome in varying degrees of anemia. Int J Gynecol Obstet 79: 93–100 10.1016/s0020-7292(02)00225-4 12427391

[bib5] Petridou E, Trichopoulos D, Kalapothaki V, Pourtsidis A, Kogevinas M, Kalmanti M, Koliouskas D, Kosmidis H, Panagiotou JP, Piperopoulou F, Tzortzatou F (1997) The risk profile of childhood leukaemia in Greece: a nationwide case–control study. Br J Cancer 76: 1241–1247 9365177 10.1038/bjc.1997.541 PMC2228112

[bib6] Roman E, Ansell P, Bull D (1997) Leukaemia and non-Hodgkin's lymphoma in children and young adults: are prenatal and neonatal factors important determinants of disease? Br J Cancer 76: 406–415 9252212 10.1038/bjc.1997.399 PMC2224068

[bib7] Roman E, Simpson J, Ansell P, Lightfoot T, Mitchell C, Eden TO (2005) Perinatal and reproductive factors: a report on haematological malignancies from the UKCSS. Eur J Cancer 41: 749–759 15763652 10.1016/j.ejca.2004.11.006

[bib8] Spector LG, Xie Y, Robison LL, Heerema NA, Hilden JM, Lange B, Felix CA, Davies SM, Slavin J, Potter JD, Blair CK, Reaman GH, Ross JA (2005) Maternal diet and infant leukemia: the DNA topoisomerase II inhibitor hypothesis: a report from the Children's Oncology Group. Cancer Epi Biom Prev 14: 651–655 10.1158/1055-9965.EPI-04-0602 15767345

[bib9] Steer P, Alam MA, Wadsworth J, Welch A (1995) Relation between maternal haemoglobin concentration and birth weight in different ethnic groups. BMJ 310: 489–491 7888886 10.1136/bmj.310.6978.489 PMC2548871

[bib10] Steer PJ (2000) Maternal hemoglobin concentration and birth weight. Am J Clin Nutr 71(Suppl): 1285S–1287S 10799403 10.1093/ajcn/71.5.1285s

[bib11] World Health Organization Expert Committee (1965) Nutrition In pregnancy and lactation; report of a WHO Expert Committee. World Health Organ Tech Rep Ser 302: 5–19 14285099

